# From climate zone to microhabitat—environmental factors affecting the coastal distribution of tiger beetles (Coleoptera: Cicindelidae) in the south-eastern European biodiversity hotspot

**DOI:** 10.7717/peerj.6676

**Published:** 2019-04-09

**Authors:** Radomir Jaskuła, Mateusz Płóciennik, Axel Schwerk

**Affiliations:** 1 Department of Invertebrate Zoology and Hydrobiology/Faculty of Biology and Environmental Protection, University of Lodz, Łódź, Poland; 2 Laboratory of Evaluation and Assessment of Natural Resources, Faculty of Horticulture, Biotechnology and Landscape Architecture, Warsaw University of Life Sciences-SGGW, Warsaw, Poland

**Keywords:** Coleoptera, Cicindelidae, Black Sea Coast, Balkan Peninsula, Habitat specialisation, Bioindicators

## Abstract

**Background:**

Tiger beetles (Coleoptera: Cicindelidae) are predatory insects usually occurring in various sandy habitats. In south-eastern Europe, especially in lowland areas located close to the sea coast, the diversity of Cicindelidae is one of the highest in the Palaearctic realm. Although previous studies conducted in different areas of the world show that many species are habitat specialists, unfortunately little is known about environmental factors affecting the diversity and distribution of tiger beetles in this region.

**Material and Methods:**

Habitat preferences for 12 tiger beetles taxa were analysed. Over 100 samples collected in eight countries located in coastal areas of the Black and Mediterranean Seas were studied, for which climate data, macrohabitat types, and soil parameters (soil humidity, salinity, pH, and structure) were investigated.

**Results:**

Most studied Cicindelidae were characterised by narrow or very narrow habitat specialisation and did not co-occur with other ones, including 11 taxa found as habitat specialists occurring only in one or two types of macrohabitat. The most eurythopic species was *Calomera littoralis nemoralis* which occupied four macrohabitat types. The climatic zone, altitude, and humidity were found as the most important factors in the distribution of the studied tiger beetle species. Salt marshes and sandy sea beaches were noted as the most diverse macrohabitat types.

**Discussion:**

Tiger beetle fauna of south-eastern Europe consists mainly of habitat specialists sensitive to environmental changes, which makes these beetles perfect bioindicators. Moreover, as a great number of studied Cicindelidae taxa occur in habitats which are under a significant human impact, we suggest that in the studied area the group can be successfully used as a flagship taxon for insect and nature conservation.

## Introduction

Tiger beetles (Cicindelidae Latreille, 1806) are an insect family (López-López & Vogler, 2017) with a worldwide distribution except for polar regions and some oceanic islands (Cassola & Pearson, 2000; Pearson & Vogler, 2001). The group includes approximately 2,800 species (Pearson & Cassola, 2005; Zettel & Wiesner, 2018) of both epigeic and arboreal, small to medium-sized beetles which are known as active predators hunting mainly for small arthropods (Pearson & Vogler, 2001; Rewicz & Jaskuła, 2018). Although the number of studies focused on habitat preferences of tiger beetles is rather limited, generally it is known that many typically terrestrial tiger beetles usually prefer various sandy habitats where both larvae and adult beetles live. Moreover, previous studies suggest that most Cicindelidae can be characterised by narrow or even very narrow habitat specialisation, and as a result they can be found only in one or at most in a few very similar types of macrohabitats (Freitag, 1979; Knisley & Pearson, 1984; Pearson, 1984; Ganeshaiah & Belavadi, 1986; Schultz & Hadley, 1987; Acciavatti & Pearson, 1989; Pearson, Barraclough & Vogler, 1997; Jaskuła, 2011, 2015). As a result, tiger beetles are regularly used as bioindicators for determining both regional and global patterns of biodiversity and have become a very important global flagship group for beetle and insect conservation (Schultz, 1989; Knisley & Hill, 1992; Pearson & Cassola, 1992, 2005; Kitching, 1996; Carroll & Pearson, 1998a, 1998b; Rodríguez, Pearson & Barrera, 1998; Andriamampianina et al., 2000; Pearson & Vogler, 2001; Zerm & Adis, 2001; Arndt, Aydin & Aydin, 2005; Brust, Hoback & Knisley, 2005; Jaskuła, 2011, 2015; Dangalle, 2013; Dangalle, Pallewatta & Vogler, 2013, 2014).

Many Cicindelidae species occupy the same areas as their larvae, which are ground-dwelling insects spending all the time from egg to pupae in burrows usually built in different types of sandy soil. Consequently, habitats, especially the parameters of soil including its structure and moisture as well as temperature and vegetation cover (often depending on climate), play an important role in the tiger beetle distribution (Pearson, 1988). As a result, significantly higher diversity and species richness of Cicindelidae are noted in tropical regions than in temperate zones (Pearson & Cassola, 1992; Cassola & Pearson, 2000) and in lowland areas (where a larger mosaic of sandy habitats can be found) than in the highlands and mountains (Pearson & Cassola, 1992; Jaskuła, 2011, 2015).

The south-eastern part of Europe, including the coastal zones of the Mediterranean, Black, and Azov Seas, is known as a very important terrestrial Pleistocene glacial refugium, both on the local (Kryštufek & Reed, 2004) and whole Western Palaearctic scale (Hewitt, 1996, 1999; Thompson, 2005; Blondel et al., 2010; Habel et al., 2010). It is also perceived as part of one of the 25 most important world biodiversity hot spots (Myers et al., 2000; Cuttelod et al., 2008). In the case of Cicindelidae, the area can be characterised by high species richness (over 40% of European species) explained by a high level of heterogeneity of sandy habitats preferred both by larvae and adults of tiger beetles and located mainly in the coastal zones of the Mediterranean, Black and Azov Seas (Putchkov & Matalin, 2003; Jaskuła, 2011).

Taking into account present knowledge concerning habitat preferences of Cicindelidae from different regions of the world as well as previous studies by the first author focused on the diversity and distribution of tiger beetle species in south-eastern Europe, the paper aims to test the following hypotheses:

1. In the studied area, tiger beetles are characterised by more or less narrow macrohabitat/microhabitat specialisation;
2. Particular tiger beetle species prefer similar types of habitat in different regions of its distributional area;
3. Occurrence of different tiger beetle species in particular regions/habitats of the studied area is correlated with the parameters of soil, particularly its humidity, pH, salinity, and structure.

## Material and Methods

### Field sampling

Adult tiger beetle species were collected by entomological hand nets during the TB-Quest Expeditions organised by the first author to the Balkan Peninsula and the Black Sea coast in 2009−2012. In total, 114 samples from Albania, Bulgaria, Greece, Romania, Macedonia FYR, Montenegro, Moldova, and Ukraine were collected for which location, GPS co-ordinates, and date were noted (Table 1; Dataset S1). A total of 12 tiger beetle taxa were collected: *Calomera aulica aulica* (Dejean, 1822), *C. fischeri fischeri* (Adams, 1817), *C. littoralis nemoralis* (Olivier, 1790), *Cephalota besseri besseri* (Dejean, 1822), *C. chiloleuca* (Fischer Von Waldheim, 1820), *C. circumdata circumdata* (Dejean, 1822), *Cicindela maritima kirgisica* Mandl, 1936*, C. monticola rumelica* Apfelbeck, 1909, *Cylindera germanica germanica* (Linnaeus, 1758), *C. trisignata hellenica* Cassola, 1973, *C. trisignata trisignata* (Dejean, 1822), and *Myriochila melancholica* (Fabricius, 1798). Adult beetles were collected during sunny hours when the activity of most tiger beetle species is the highest. Although we noted presence of tiger beetle larvae in almost all sampling sites, it was not possible to recognise if larve of all noted Cicindelidae species were present in the particular habitat, as in case of some taxa studied by us, their juvenile stages are still unknown. As a consequence we decided to study only adult beetles.

**Table 1:** Sampe codes and GPS co-ordintates.

Sample code	GPS co-ordinates	Sample code	GPS co-ordinates
AL-02/T3B-2010	N41.16325 E20.22933	GR-29/T32-2011	N36.80451 E22.69421
AL-04A/T5A-2010	N41.77051 E19.60032	GR-30/T33-2011	N40.82814 E25.97922
AL-06/T7-2010	N41.57539 E19.47552	GR-31/T34-2011	N41.00154 E25.16867
AL-07/T8-2010	N41.12435 E19.44858	MD-01/T1-2012	N46.91151 E28.39807
AL-08A/T9A-2010	N40.90978 E19.41322	MK-01A/T3A-2010	N40.94522 E20.90385
AL-08B/T9B-2010	N40.90978 E19.41322	MNE-01/T1-2011	N42.16319 E19.22248
AL-08C/T9C-2010	N40.90978 E19.41322	MNE-02/T2-2011	N41.87111 E19.33309
AL-09/T10-2010	N40.98621 E19.49688	RO-03/T2-2012	N45.02893 E29.16031
AL-10/T11-2010	N40.74978 E19.57787	RO-04/T3-2012	N44.90882 E28.83239
AL-12/T13-2010	N40.62849 E19.34299	RO-05/T4-2012	N44.88047 E28.80822
AL-13/T3-2011	N41.86185 E19.44742	RO-06/T5-2012	N44.78225 E28.90062
AL-14A/T4A-2011	N41.71029 E19.60026	RO-07A-T6A-2012	N44.62350 E28.79436
AL-14B/T4B-2011	N41.71029 E19.60026	RO-07B/T6B-2012	N44.61967 E28.30752
AL-15/T5-2011	N41.74930 E19.57265	RO-08/T7-2012	N44.67431 E28.89582
AL-16/T6-2011	N41.75259 E19.59838	RO-09/T8-2012	N44.53820 E28.72625
AL-17/T7-2011	N41.68125 E19.67219	RO-10/T9-2012	N44.37732 E28.71192
AL-18/T8-2011	N41.59049 E19.58026	RO-11/T10-2012	N44.44574 E28.73505
AL-19/T9-2011	N40.98786 E19.48419	RO-12/T11-2012	N44.43155 E28.77055
AL-20/T10-2011	N40.98268 E19.49548	RO-13/T12-2012	N44.44422 E28.74371
AL-21/T11-2011	N40.67619 E19.33409	RO-14/T21-2012	N45.34997 E26.69501
AL-22/T12-2011	N40.67309 E19.35832	RO-15/T22-2012	N46.60329 E23.79886
AL-23/T13-2011	N39.74292 E20.00576	UA-01/T1-2011	N47.09562 E38.18540
AL-24/T14-2011	N39.69515 E20.11696	UA-02A/T2A-2011	N47.07738 E38.12819
BG-02/T13-2012	N42.02339 E28.00734	UA-02B/T2B-2011	N47.07738 E38.12819
BG-03/T14-2012	N42.06318 E27.97311	UA-03A/T3A-2011	N47.09569 E38.01092
BG-04/T15-2012	N42.10304 E27.92366	UA-03B/T3B-2011	N47.09569 E38.01092
BG-05/T16-2012	N42.14655 E27.87794	UA-04/T4-2011	N47.08143 E37.69160
BG-06/T17-2012	N42.34988 E27.72104	UA-05/T5-2011	N46.94367 E37.38399
BG-07/T18-2012	N42.55187 E27.48438	UA-06/T6-2011	N46.87361 E37.30523
BG-08/T19-2012	N43.19124 E27.73240	UA-07/T7-2011	N46.70605 E36.83310
BG-09/T20-2012	N43.57218 E28.58338	UA-08/T8-2011	N46.66028 E36.29574
GR-01/T14-2010	N39.18940 E20.53221	UA-09/T9-2011	N46.65631 E35.34950
GR-03/T16-2010	N39.95805 E22.69696	UA-10/T10-2011	N46.74554 E35.35399
GR-04/T17-2010	N40.15725 E22.54858	UA-11/T11-2011	N46.53403 E35.10083
GR-05/T18-2010	N40.29430 E22.61182	UA-12/T12-2011	N46.50789 E35.11976
GR-06/T19-2010	N40.65620 E23.16222	UA-13A/T13A-2011	N46.16808 E34.78357
GR-07/T20-2010	N40.78218 E23.82907	UA-13B/T13B-2011	N46.16808 E34.78357
GR-08/T21-2010	N40.89414 E24.85901	UA-14/T14-2011	N46.15247 E34.60260
GR-11/T24-2010	N40.82156 E25.98921	UA-15/T15-2011	N45.20258 E35.64101
GR-12/T15-2011	N39.03415 E20.76072	UA-16/T16-2011	N45.40428 E35.88406
GR-13/T16-2011	N38.67767 E20.93182	UA-17/T17-2011	N45.97222 E33.72799
GR-14/T17-2011	N38.41763 E21.36271	UA-18A/T18A-2011	N46.60185 E32.11789
GR-15/T18-2011	N38.37430 E21.55359	UA-18B/T18B-2011	N46.60185 E32.11789
GR-16/T19-2011	N38.18333 E21.39320	UA-19A/T19A-2011	N46.83873 E31.58393
GR-17/T20-2011	N38.15959 E21.38517	UA-19B/T19B-2011	N46.83873 E31.58393
GR-18/T21-2011	N38.15549 E21.36802	UA-20/T20-2011	N46.63146 E31.37901
GR-19A/T22A-2011	N37.99217 E21.27229	UA-21/T21-2011	N46.57421 E30.75625
GR-19B/T22B-2011	N37.99217 E21.27229	UA-22A/T22A-2011	N45.48260 E29.14931
GR-20/T23-2011	N37.64011 E21.47691	UA-22B/T22B-2011	N45.48260 E29.14931
GR-21/T24-2011	N37.61262 E21.45359	UA-23/T23-2011	N45.44802 E29.44001
GR-22/T25-2011	N37.51818 E21.59096	UA-24A/T24A-2011	N45.53664 E29.65798
GR-23/T26-2011	N36.95367 E21.69121	UA-24B/T24B-2011	N45.53664 E29.65798
GR-24/T27-2011	N36.95252 E21.66309	UA-25A/T25A-2011	N45.74124 E29.78605
GR-25/T28-2011	N37.05459 E22.45243	UA-25B/T25B-2011	N45.74124 E29.78605
GR-26/T29-2011	N36.80451 E22.69421	UA-26A/T26A-2011	N45.90452 E30.11342
GR-27/T30-2011	N36.66068 E23.02514	UA-26B/T26B-2011	N45.90452 E30.11342
GR-28/T31-2011	N36.78868 E23.07484		

**Note:**

AL, Albania; BG, Bulgaria; GR, Greece; MD, Moldova; MK, Macedonia FYR; MNE, Montenegro; RO, Romania; UA, Ukraine. Numbers at the end of sample code indicate year of collecting.

Sampling sites were located in climatic zones accepted after Beck et al. (2018), while every tiger beetle habitat was classified to one of the macrohabitat types distinguished earlier by the first author (Jaskuła, 2011). Moreover, at every sampling site, soil pH and soil humidity (%) were measured in three places where tiger beetles were observed and average values of those measurements were noted. Additionally, three sub-samples of soil for further laboratory analysis were collected (in total 150 ml of volume) in the same places of the sampling site where pH and humidity of soil were measured.

### Laboratory analysis

To check the soil structure, all samples were dried separately on Petri dishes in an electronic drier. Next, every sample was weighed, and after that it was sifted on electronic sieves. All received parts of soil particles gravel (>2 mm), sand (0.0632 mm), silt (0.063–0.002 mm), and clay (≤0.002 mm) were weighed. To estimate the proportion of particular soil particles in each sample, the values of their weight were compared with the total weight of the entire soil sample.

To check soil salinity, in the case of every sample, a volume of two ml of soil was dissolved in 100 ml of distilled water. Then, using the WTW Multi 350i probe, electrical conductivity of soil-water solution was measured (three measurements were done to note the average value used in further analysis).

### Statistical methods

Statistical analysis concerns 112 samples of 11 Cicindelidae taxa (presence/absence data): *Calomera aulica aulica* (Caa), *C. fischeri fischeri* (Cff), *C. littoralis nemoralis* (Cln), *Cephalota besseri besseri* (Cbb), *C. chiloleuca* (Cch), *C. circumdata circumdata* (Ccc), *Cicindela monticola rumelica* (Cmr), *Cylindera germanica germanica* (Cgg), *C. trisignata hellenica* (Cth), *C. trisignata trisignata* (Ctt), and *Myriochila melancholica* (Mm). Species *Cicindela maritima kirgisica* (Cmk) and samples AL-02 and UA-03 were excluded from macrohabitat analysis due to the fact that they were outliers in the analysed communities. This material was supplemented by data on: 1/five microhabitat environmental parameters—altitude, soil pH, soil humidity, soil salinity, and soil sediment granulometry (percentage share of gravel, sand, silt, and clay), 2/five macrohabitats types—saltmarshes, banks of rivers, banks of lakes, sandy sea beaches, sandy-stony sea beaches, and 3/four climatic zones: arid-steppe-cold (Bsk), temperate with dry, hot summer (Csa), temperate with no dry season and hot summer (Cfb), and cold without any dry season and with warm summer (Dfb).

Multivariate statistics were calculated for biotic and environmental data. Principal Component Analysis (PCA) on normalised data was conducted for the environmental ordination of sites investigated, divided into two geographical regions—A (Black Sea Basin) and B (Mediterranean Sea Basin). According to Non-metric Multidimensional Scaling (calculated using presence/absence transformed data, the Bray–Curtis similarity index, and 50 restarts, excluding outlier samples UA-03 and AL-02), tiger beetle samples were divided into four climatic zones: Bsk, Csa, Cfb, and Dfb. Taxa characteristic of each of four zones and dissimilarity between those community types were obtained using the SIMPER analysis with Bray–Curtis similarity and cut-off for low contributions 100%. Detrended Canonical Correspondence Analysis (DCCA) was implemented to recognise data distribution (linear or unimodal) with detrending by segments. As the length of DCCA gradient was 1.5 SD units for the first axis and 1.58 for the second DCCA axis, redundancy analysis (RDA) with scaling focused on inter-species correlation and species scores divided by standard deviation was conducted to recognise the main environmental factors determining species occurrence. To test the significance of environment-species relation, the unrestricted Monte Carlo Permutation Test was applied with automatic selection under the full model for all environmental variables. Statistical analyses were performed using PRIMER 6 and Canoco 4.5 software (Clark & Gorley, 2001; Ter Braak & Šmilauer, 2002).

## Results

### Macrohabitat preferences

**Figure 1:**
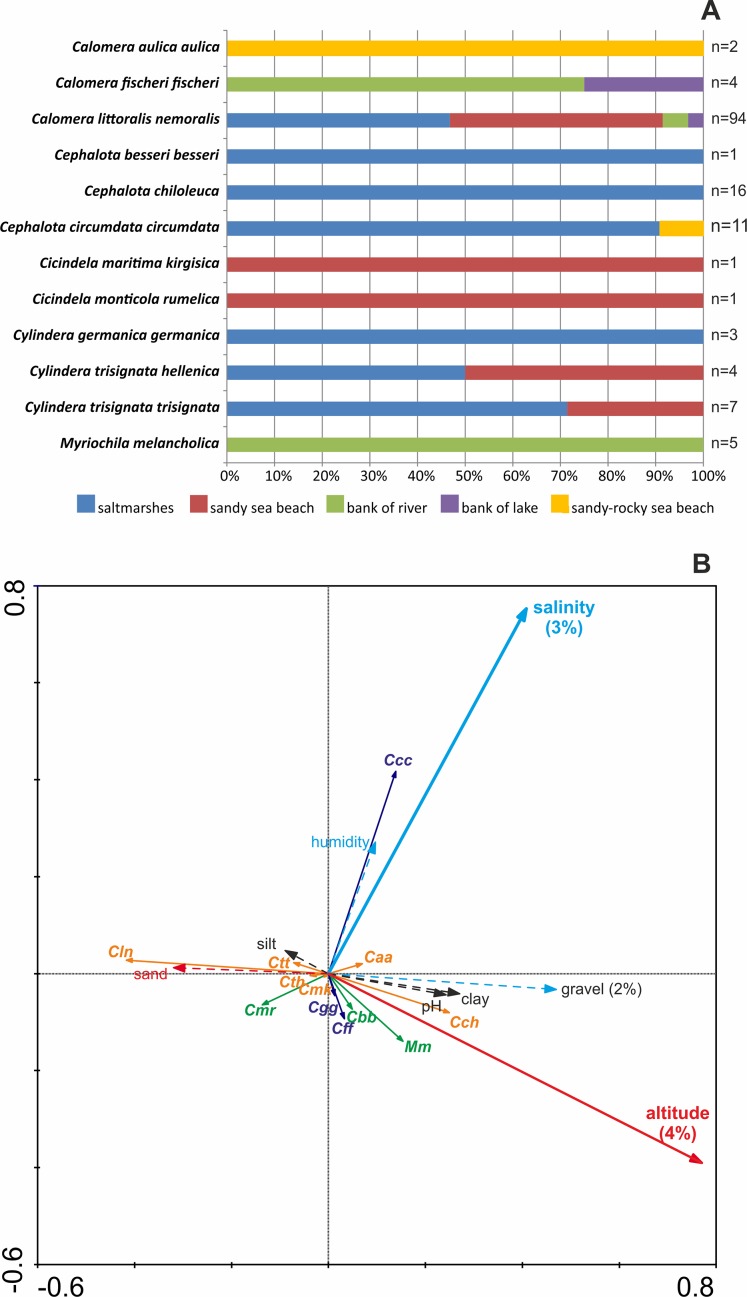
Distribution of recorded Cicindelidae species along investigated macrohabitats (A) and gradients of investigated physico-chemical parameters (B). (A) Colours indicate macrohabitats where one or another species were recorded, *n—*number of sites where species was noted; (B) results of RDA analysis; thick arrows—factors significantly explaining species distribution, dashed arrows—insignificant factors; red arrows—factors correlated with Axis 1, light blue one—factors correlated with Axis 2, black—factors not correlated with Axes 1 and 2; percent value below factor name—variance explained; taxa marked by orange colour—correlated mostly with Axis 1, taxa marked by navy blue colour—correlated mostly with Axis 2, taxa marked by green colour—correlated parallel with Axis 1 and Axis 2.

In the study, 12 Cicindelidae taxa were recorded in five different macrohabitat types. Eight tiger beetle species, *Calomera aulica aulica*, *Cephalota besseri besseri*, *C. chiloleuca*, *C. circumdata circumdata*, *Cicindela maritima kirgisica*, *C. monticola rumelica*, *Cylindera germanica germanica,* and *Myriochila melancholica*, were noted only in one macrohabitat type. *Calomera fischeri fischeri*, *Cylindera trisignata hellenica*, and *C. t. trisignata* occurred in two macrohabitats, while the most opportunistic species was *Calomera littoralis nemoralis*, which was noted in four different habitats. On the other hand, the highest diversity of Cicindelidae was recorded in saltmarshes (eight taxa or 67% of studied fauna) and on sandy sea beaches (five species or 42% of studied fauna). River banks, lake shores, and sandy-rocky sea beaches were characterised only by one to three tiger beetle species (respectively 25%, 17%, and 8% of noted fauna; Fig. 1A).

### Community structure and environment

**Figure 2:**
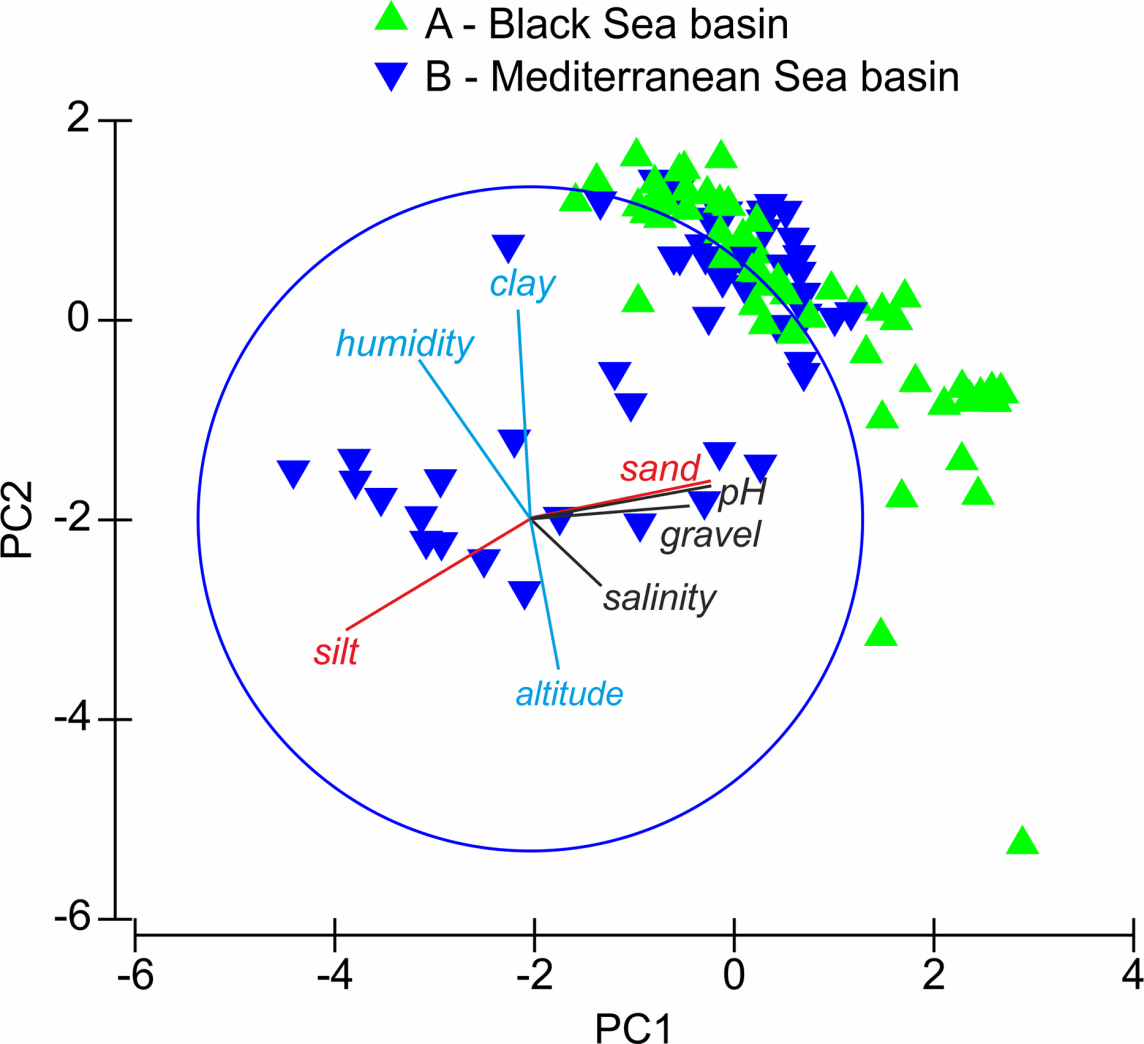
Results of PCA analysis. Triangles A and B—sites investigated; red lines—factors correlated with axis PC1, blue lines—factors correlated with axis PC2, black lines—factors not correlated with PC1 and PC2 axes.

The distribution of the sites in the PCA (Fig. 2) indicates that sites from group A are positively or weakly negatively correlated with PC Axis 1 and show moderate, indirect variation according to Axis 2. Sites from group B are negatively correlated or not correlated to PC Axis 1 and Axis 2. The correlation of the factors with the ordination axis shows that study sites in the Black Sea basin are often located on sandy soils with higher pH values and gravel content and reveal weaker altitudinal and humidity patterns, whereas those in the Mediterranean Sea basin are often located on silty soils and reveal higher altitudinal and humidity patterns. The silt and sand content and the pH value are the most important factors differentiating sites from groups A and B.

However, the RDA indicates that sediment composition does not have a significant influence on the studied beetle communities. Redundancy analysis Axis 1 represents 6.1% of species variance and as much as 49.4% of species-environment relation variance. Redundancy analysis Axis 2 represents 3% of species variance and 24.3% of species-environment relation variance. Among environmental variables, altitude (*P* = 0.002) and soil salinity (*P* = 0.002) are significant factors and explain 4% and 3% of total variance of species distribution among the samples respectively. The gravel content can be regarded as almost a significant factor (*P* = 0.064) explaining 2% of total variance of species distribution among the samples. The altitude and gravel content are positively correlated with Axis 1, the share of sand fraction is negatively correlated with Axis 1, whereas soil salinity and humidity are positively correlated with Axis 2. The silt and clay share in the sediment and pH are not correlated with canonical Axis 1 or Axis 2. Results of RDA indicate (Fig. 1B) that *Cephalota circumdata circumdata* prefers higher soil salinity and humidity. *Calomera littoralis nemoralis* prefers sandy soil and low amounts of gravel and clay, as well as low pH and also sites of low altitude (characteristics of sites near sea beaches), whereas *C. chiloleuca* and *M. melancholica* appear at sites of higher altitude (sites more distant from the sea shore). Other tiger beetle taxa (*Calomera aulica aulica*, *Cephalota besseri besseri*, *Calomera fischeri fischeri*, *Cylindera germanica germanica*, *Cicindela monticola rumelica*, *C. maritima kirgisica*, *C. trisignata hellenica*, and *C. t. trisignata*) are weakly correlated with factors of Axis 1 and Axis 2, correlated parallel with Axis 1 and Axis 2 or not correlated with the factors measured.

**Figure 3:**
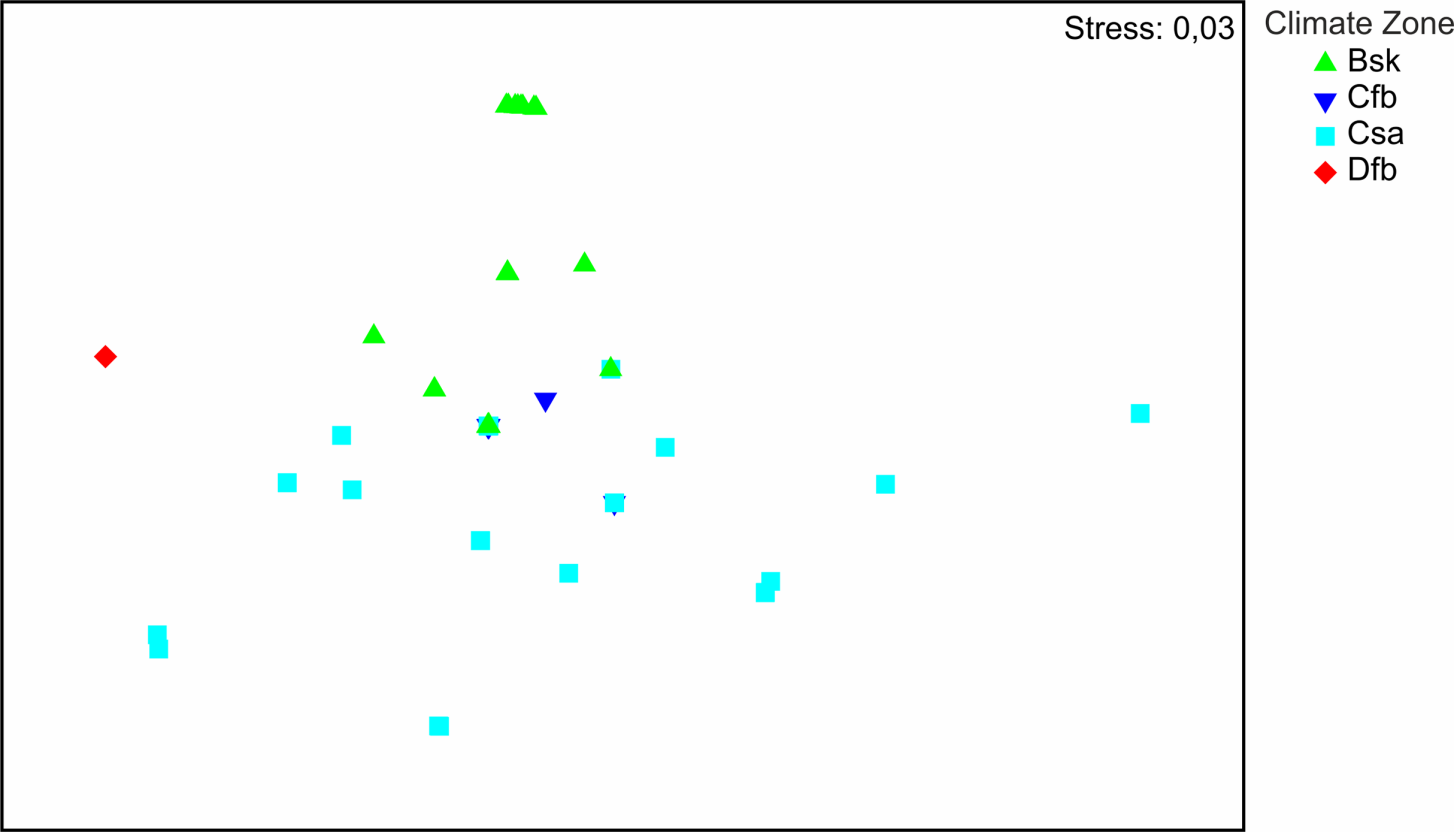
Results of NMDS. Symbols represent tiger beetle communities of sites located in climate zones: Bsk (arid-steppe-cold), Cfb (temperature with no dry season and hot summer), Csa (temperate with dry, hot summer), Dfb (cold without any dry season and with warm summer).

The distribution of the recorded species on a large geographical scale follows two main climatic zones: Bsk (arid-steppe-cold) and Csa (temperate with dry and hot summer) (Fig. 3). Csa communities are much more diverse than Bsk communities. The SIMPER analysis (App. 1) shows that faunas of Csa and Bsk zones are separate except for *Calomera littoralis nemoralis*, which is distributed in three climatic zones including Cfb, which is intermediate between the Csa and Bsk zones. The taxa distributed solely in the Bsk zone are *Cephalora chiloleuca* and *Cylindera germanica germanica*. Taxa which are distributed in the Csa but not in the Bsk zone are *Cephalota circumdata circumdata*, *Cylindera trisignata trisignata*, *Myriochila melancholica*, *Calomera fischeri fischeri*, and *C. aulica aulica*. *Cylindera trisignata hellenica* appears in both zones Bsk and Csa. The Dfb zone, represented by only one species (*Calomera littoralis nemoralis*) recorded from one site (MK-01), needs to be excluded from the SIMPER analysis.

## Discussion

### Geographical and climatic gradients vs. Cicindelidae diversity and distribution

As in the case of many insect groups, the distribution and diversity of Cicindelidae are strictly connected with a geographical region of the world, climate and weather conditions, as well as a habitat type (Pearson & Cassola, 1992; Pearson, 1988; Pearson & Vogler, 2001). Generally it is known that the number of tiger beetle species occurring in warm tropical regions is significantly higher than in temperate zones and that lowland areas are characterised by higher species richness than the highlands and mountains (Pearson & Cassola, 1992; Acciavatti & Pearson, 1989; Andriamampianina et al., 2000; Pearson & Vogler, 2001; Jaskuła & Rewicz, 2015; Jaskuła, Rewicz & Kwiatkowski, 2015). This is due to the fact that such regions are characterised by high average annual temperatures, usually higher air humidity and a significantly larger mosaic of sandy habitats which are attractive for many Cicindelidae species (Pearson & Vogler, 2001). In the case of our study area, the diversity and species richness of tiger beetles occurring in the lowlands, especially in the Mediterranean and Black Seas coastal zones, clearly confirm this general worldwide tendency as about 40% of European Cicindelidae fauna is known from this area (Putchkov & Matalin, 2003; Jaskuła, 2011). Although it was not possible to visit all places potentially attractive for tiger beetles in the area, and as a result we were not able to study all species occurring in this region, and moreover, for some of the analysed taxa it was possible to use only single samples, we can note that the composition of Cicindelidae fauna within the study area is significantly changing along the geographical (Fig. 2) and also climatic gradients (Fig. 3). That was observed even if the material had been collected over a few years but only during summer months, which simply excluded the possibility of noting tiger beetle species with a different type of phenological activity (Willis, 1967; Schultz, 1989; Knisley, Schultz & Hasewinkel, 1990). The warmest climate zone included in our study (temperate with dry and hot summer) known from the south-eastern Balkan Peninsula (occurring mainly along the sea coast of Montenegro, Albania, and Greece) was characterised by the highest species richness compared to the part of the Bulgarian Black Sea coast (temperate with no dry season and a hot summer climatic zone) and the Ukrainian Black Sea coast (arid-steppe-cold climate zone). Such distribution of species richness is known from the literature as the latitudinal diversity gradient and was recorded for many plant and animal taxa all around the world (Gaston, 2000; Willig, Kaufmann & Stevens, 2003; Pimm & Brown, 2004; Cardillo, Orme & Owens, 2005). Although there is still a lack of such studies upon tiger beetle faunas in the case of some continents, such a diversity gradient can be observed also in this insect group (Pearson & Cassola, 1992).

The differences in tiger beetle faunas between particular regions distinguished within the studied area (Fig. 2) can be probably explained also by geological history of south-eastern Europe as it was shown on the basis of molecular data for *Calomera littoralis* occurring both on the Mediterranean and Black Seas coasts (Jaskuła et al., 2016). In the paper, it was demonstrated that Pleistocene glaciations and associated sea level changes in the Mediterranean/Pontic region (including contemporary isolation of waters of the present day Black and Mediterranean Seas) had a profound effect on the genetic diversity and distribution of this widely distributed coastal tiger beetle species, generating a significant level of diversity within this taxon. A disconnection of the Mediterranean and Pontic basins which was present from ca. 2 to ca. 1.5 Ma as a result of such sea level fluctuations, turned the Meothic Sea, one of several predecessors of the Black Sea, into the predominantly freshwater Pontos Sea/Lake (Grinevetsky et al., 2015). Significant changes in salinity, and as a result also in the parameters of soils located on the sea coasts between both water reservoirs, were possible mainly because of different river systems located in the Black and Mediterranean Seas basins. In the case of the first one, large rivers were flowing waters to the isolated Pontos Sea (and are still flowing them to the Black Sea), which resulted in decline in salinity of both sea waters and soils on the sea coast. Moreover, large rivers bring large volumes of sediments that are often deposited close to the sea coast, which can possibly influence the soil structure in sandy habitats attractive for tiger beetles. On the other hand, the Mediterranean Sea basin of the present-day Balkan Peninsula can be characterised by a lack of such large rivers. There are mainly small streams as well as small and medium-sized rivers often flowing waters with small volumes of sediments collected directly from the mountain areas (Allen, 2000; Yanko-Hombach et al., 2006; Blondel et al., 2010).

### Macro- and microhabitat preferences

As in the case of many epigeic Cicindelidae species, adult beetles occupy the same areas as their larvae, except the climate conditions, the structure of microhabitat, especially soil components, play an important role in their general distribution patterns (Pearson & Vogler, 2001) as well as in microhabitat segregation by particular taxa (Ganeshaiah & Belavadi, 1986; Schultz & Hadley, 1987; Knisley & Hill, 1992; Satoh & Hori, 2005). In tiger beetles, the soil parameters, including soil composition, moisture, chemistry, and temperature, are tested at least by females after copulation and before laying eggs in the soil. Such behaviour allows them to choose the optimal microhabitat type which can increase their reproductive success (Pearson & Vogler, 2001; Brust, Hoback & Knisley, 2005).

Our results show that almost all of the studied Cicindelidae taxa (92% of investigated fauna) are habitat specialists occurring in only one to two macrohabitat types, with *Calomera littoralis nemoralis* as the only exception due to the fact that the species was noted from almost all studied macrohabitat types (Fig. 1). Wide habitat preferences of *C. littoralis nemoralis* found in the presented study confirm earlier literature data as it was noted as the most eurythopic tiger beetle species according to the habitat type in the Balkan Peninsula (Jaskuła, 2011). Moreover, it is also known as the only Cicindelidae species in the studied area with opportunistic vegetarian behaviour, which can also promote a wide habitat distribution observed in this insect (Jaskuła, 2013). On the other hand, in the case of at least four species (*Calomera aulica aulica*, *Cephalota besseri besseri*, *Cicindela maritima kirgisica*, and *C. monticola rumelica*), for which only single samples were available, we can only speculate if they are really habitat specialists or can be found in different (e.g. not checked in this study) types of macrohabitats. Definitely more data are necessary to confirm macrohabitat preferences of these taxa, especially since all of them are known as rare or even very rare in the studied area (often with a very restricted distributional area) (Putchkov & Matalin, 2003, Franzen, 2006; Matalin, 1999; Jaskuła, 2011). Although additional data are needed to clarify habitat preferences of some of the Cicindelidae species studied by us, our results clearly confirm narrow or very narrow habitat specialisation observed as typical of tiger beetles in different regions of the world (Freitag, 1979; Knisley, 1984; Knisley & Pearson, 1984; Pearson, 1984; Schultz & Hadley, 1987; Ganeshaiah & Belavadi, 1986; Zerm & Adis, 2001; Satoh & Hori, 2005; Satoh et al., 2006; Jaskuła, 2011, 2015; Rodríguez-Flores et al., 2016). For example, on the basis of data summarised by Pearson, Barraclough & Vogler (1997) and available for all North American Cicindelidae, it was found that only *Cicindela tranquebarica* occurred in as many as six habitat categories, even if 17 different habitat types were recognised. Moreover, in the USA, in Sulphur Springs Valley (Arizona) only *Cicindelidia nigrocoerulea*, one of 20 species recorded during the studies, was noted in more than one habitat type (Knisley & Pearson, 1984), while in the Colfax County (New Mexico) only four of 19 species (*Cicindela fulgida*, *C. tranquebarica*, *Cicindelidia punctulata*, and *C. nigrocoerulea*) were found as habitat generalists occurring in seven different macrohabitat types (Knisley, 1984). Similar results were provided also from Asia, both by Acciavatti & Pearson (1989), from the Indian subcontinent, where among Cicindelidae taxa only *Calochroa flavomaculata* was recorded from several different habitat types, as well as by Satoh et al. (2006) from Japan, where only *Cicindela transbaicalica* was distributed widely along the river in the Tedori River System (two other studied species were habitat specialists). Narrow habitat specialisation was found also in tiger beetles occurring in Australia (Freitag, 1979), where among 29 studied species only *Myriochila mastersi* and *M. semicincta* were found as habitat generalists, South America (Pearson, 1984), where *Odontocheila annulicornis* was the only one cicindelid taxon (of 29 species) recorded in more than one forest habitat type in the Tambopata Reserve Zone, Peru, as well as in North Africa, where among four studied tiger beetle species only *Lophyra flexuosa* was noted as eurytopic and occurred in four macrohabitat types (Jaskuła, 2015).

Numerous literature data from many regions of the world show that different soil parameters play a very important role for epigeic Cicindelidae (for review see Pearson & Vogler, 2001). Although in our study we were not able to provide a large number of samples for all studied tiger beetle taxa (Fig. 1), and as a consequence, it was not possible to estimate any key factor in the case of microhabitat parameters for such beetles, we still can find that the occurrence of a few of them is connected with some of the measured microhabitat parameters. For example, *Calomera littoralis nemoralis*, recognised in the study as the most eurythopic species according to the macrohabitat type, is also a taxon which strongly prefers lower elevations. On the other hand, *Cephalota circumdata circumdata* was found as a species strongly preferring soils with higher salinity values, which of course is very characteristic of salt marshes.

It is important to note that among all studied macrohabitat types salt marshes and sandy sea beaches were characterised by the highest species richness (respectively seven species or 58% of fauna and five species or 42% of fauna) (Fig. 1). High importance of such habitats for Cicindelidae was earlier noted also in many other areas in the Mediterranean region (Šekeroğlu & Aydõn, 2002; Arndt, Aydin & Aydin, 2005; Aydõn, 2011; Jaskuła, 2015; Rodríguez-Flores et al., 2016; Assmann et al., 2018). On the other hand, such types of habitat are known as threatened in great parts of Europe and all the Mediterranean region, mainly as a result of human activity (Davy, Bakker & Figueroa, 2009), including tourist activity and rapid development of tourist infrastructure (Arndt, Aydin & Aydin, 2005). As a consequence, based on such data, at least 75% of species noted by us in the study already are or can be potentially threatened in the near future, even if actually some of them are still common and/or abundant in the region.

## Conclusions

Narrow or even very narrow habitat specialisation noted by us in the studied Cicindelidae taxa clearly confirms the high value of this beetle group as important bioindicators and a flagship taxon for nature conservation. Although we were not able to provide large data for all studied taxa (and the study was done only on the basis of adult beetles), we believe that high sensitivity of tiger beetles to potential environmental changes, including climatic and habitat ones, makes them model organisms for biologists, ecologists, and nature conservationists who are focused not only on beetles and/or insects but also on habitat types occupied by Cicindelidae.

## Supplemental Information

10.7717/peerj.6676/supp-1 Supplemental Information 1 Sample data. Data set with raw data used in the study including GPS coordinates, environmental data, and species diversity of particular samples.

## Additional Information and Declarations

### Data Availability

The following information was supplied regarding data availability: All raw data are provided in Dataset 1. The raw data show coordinates, environmental data, and species diversity of particular samples. These data were used for all analyses presented in text.

## References

[ref-2] Acciavatti RE, Pearson DL (1989). The tiger beetle genus *Cicindela* (Coleoptera, Insecta) from the Indian subcontinent. Annals of Carnegie Museum.

[ref-3] Allen HD (2000). Mediterranean ecogeography.

[ref-4] Andriamampianina L, Kremen C, Vane-Wright D, Lees D, Razafimahatratra V (2000). Taxic richness patterns and conservation of Madagascar tiger beetles (Coleoptera: Cicindelidae). Journal of Insect Conservation.

[ref-5] Arndt E, Aydin N, Aydin G (2005). Tourism impairs tiger beetle (Cicindelidae) populations—a case study in a Mediterranean beach habitat. Journal of Insect Conservation.

[ref-6] Assmann T, Boutaud E, Buse J, Gebert J, Drees C, Friedman ALL, Khoury F, Marcus T, Orbach E, Renan I, Schmidt C, Zumstein P (2018). The tiger beetles (Coleoptera, Cicindelidae) of the southern Levant and adjacent territories: from cybertaxonomy to conservation biology. ZooKeys.

[ref-7] Aydõn G (2011). Vulnerability of *Megacephala* (*Grammognatha*) *euphratica euphratica* Latreille and Dejean, 1822 (Coleoptera: Cicindelidae) in natural and disturbed saltmarsh and salt meadow habitats in Turkey. African Journal of Biotechnology.

[ref-8] Beck HE, Zimmermann NE, McVicar TR, Vergopolan N, Berg A, Wood EF (2018). Present and future Köppen-Geiger climate classification maps at 1-km resolution. Scientific Data.

[ref-9] Blondel J, Aronson J, Bodiou J-Y, Boeuf G (2010). The Mediterranean region: biological diversity through space and time.

[ref-10] Brust ML, Hoback WW, Knisley CB (2005). Biology, habitat preference, and larval description of *Cicindela cursitans* Leconte (Coleoptera: Carabidae: Cicindelinae). Coleopterists Bulletin.

[ref-11] Cardillo M, Orme CDL, Owens IPF (2005). Testing for latitudinal bias in diversification rates: an example using New World birds. Ecology.

[ref-12] Carroll SS, Pearson DL (1998a). Spatial modelling of butterfly species richness using tiger beetles (Cicindelidae) as bioindicator taxon. Ecological Applications.

[ref-13] Carroll SS, Pearson DL (1998b). The effects of scale and sample size on the accuracy of spatial predictions of tiger beetle (Cicindelidae) species richness. Ecography.

[ref-14] Cassola F, Pearson DL (2000). Global patterns of tiger beetle species richness (Coleoptera: Cicindelidae): their use in conservation planning. Biological Conservation.

[ref-15] Clark KR, Gorley RN (2001). PRIMER v5: user manual/tutorial— PRIMER-E.

[ref-16] Cuttelod A, Garcia N, Abdul Malak D, Temple H, Katariya V, Vie J-C, Hilton-Taylor C, Stuart SN (2008). The Mediterranean: a biodiversity hot spot under threat. The 2008 Review of The IUCN Red List of Threatened Species.

[ref-17] Dangalle CD (2013). The current status of the tiger beetle species of the coastal habitats of Sri Lanka. Journal of Tropical Forestry and Environment.

[ref-18] Dangalle CD, Pallewatta N, Vogler AP (2013). The association between body-size and habitat-type in tiger beetles (Coleoptera, Cicindelidae) of Sri Lanka. Ceylon Journal of Science (Biological Sciences).

[ref-19] Dangalle CD, Pallewatta N, Vogler AP (2014). Distribution and habitat preferences of tiger beetles (Coleoptera: Cicindelidae) of the riverine ecosystems of Sri Lanka. Journal of Threatened Taxa.

[ref-20] Davy AJ, Bakker JP, Figueroa ME, Silliman BR, Bertness MD, Grosholz ED (2009). Human modification of European salt marshes. Human Impact on Salt Marshes—A Global Perspective.

[ref-21] Franzen M (2006). Verbreitung und Lebensräume der Sandlaufkäfer der Peloponnes-Halbinsel, Griechenland (Coleoptera, Cicindelidae). Nachrichtenblatt der Byerischen Entomologen.

[ref-22] Freitag R (1979). Reclassification, phylogeny and zoogeography of the Australian species of *Cicindela* (Coleoptera: Cicindelidae). Australian Journal of Zoology Supplementary Series.

[ref-23] Ganeshaiah KN, Belavadi VV (1986). Habitat segregation in four species of adult tiger beetles (Coleoptera; Cicindelidae). Ecological Entomology.

[ref-24] Gaston KJ (2000). Global patterns in biodiversity. Nature.

[ref-25] Grinevetsky SR, Zonn IS, Zhiltsov SS, Kosarev AN, Kostianoy AG (2015). The Black Sea encyclopedia.

[ref-26] Habel JC, Drees C, Schmitt T, Assmann T, Habel JC, Assmann T (2010). Review: refugial areas and postglacial colonizations in the Western Palearctic. Relict Species: Phylogeography and Conservation Biology.

[ref-27] Hewitt GM (1996). Some genetic consequences of ice ages, and their role in divergence and speciation. Biological Journal of the Linnean Society.

[ref-28] Hewitt GM (1999). Post-glacial re-colonization of European biota. Biological Journal of the Linnean Society.

[ref-29] Jaskuła R (2011). How unique is the tiger beetle fauna (Coleoptera Cicindelidae) of the Balkan Peninsula?. ZooKeys.

[ref-30] Jaskuła R (2013). Unexpected vegetarian feeding behaviour of a predatory tiger beetle *Calomera littoralis nemoralis* (Olivier, 1790) (Coleoptera: Cicindelidae). Journal of the Entomological Research Society.

[ref-31] Jaskuła R (2015). The Maghreb—one more important biodiversity hot spot for tiger beetle fauna in the Mediterranean region. ZooKeys.

[ref-64] Jaskuła R, Rewicz T (2015). Tiger beetles (Coleoptera: Carabidae: Cicindelinae) of Tunisia: distribution, phenology, taxa list and new records. African Entomology.

[ref-65] Jaskuła R, Rewicz T, Kwiatkowski K (2015). Tiger beetle fauna (Coleoptera: Carabidae, Cicindelinae) of Morocco: distribution, phenology and list of taxa. Entomologica Fennica.

[ref-32] Jaskuła R, Rewicz T, Płóciennik M, Grabowski M (2016). Pleistocene phylogeography and cryptic diversity of a tiger beetle, *Calomera littoralis*, in North-Eastern Mediterranean and Pontic regions inferred from mitochondrial COI gene sequences. PeerJ.

[ref-34] Kitching IJ (1996). Identifying complementary areas for conservation in Thailand: an example using owls, hawkmoths and tiger beetles. Biodiversity and Conservation.

[ref-35] Knisley CB (1984). Ecological distribution of tiger beetles (Coleoptera: Cicindelidae) in Colfax County, New Mexico. Southwestern Naturalist.

[ref-36] Knisley CB, Hill JM (1992). Effects of habitat change from ecological succession and human impact on tiger beetles. Virginia Journal of Science.

[ref-37] Knisley CB, Pearson DL (1984). Biosystematics of larval tiger beetles of the Sulphur Springs Valley, Arizona. Transactions of the American Entomological Society.

[ref-38] Knisley CB, Schultz TD, Hasewinkel TH (1990). Seasonal activity and thermoregulatory behavior of *Cicindela patruela* (Coleoptera: Cicindelidae). Annals of the Entomological Society of America.

[ref-1] Kryštufek B, Reed JM (2004). Balkan biodiversity: pattern and process in the European hotspot.

[ref-39] López-López A, Vogler AP (2017). The mitogenome phylogeny of Adephaga (Coleoptera). Molecular Phylogenetics and Evolution.

[ref-40] Matalin AV (1999). The tiger-beetles of the *hybrida* species-group. II. A taxonomic review of subspecies of *Cicindela sahlbergii* Fischer von Waldheim, 1824 (Cooleoptera, Carabidae, Cicindelini). Advances in Carabidology.

[ref-41] Myers N, Mittermeier RA, Mittermeier CG, Da Fonseca GAB, Jennifer K (2000). Biodiversity hotspots for conservation priorities. Nature.

[ref-42] Pearson DL (1984). The tiger beetles (Coleoptera: Cicindelidae) of the Tambopata Reserved Zone, Madre e Dios, Peru. Revista Peruana de Entomologia.

[ref-43] Pearson DL (1988). Biology of tiger beetles. Annual Review of Entomology.

[ref-44] Pearson DL, Barraclough TG, Vogler AP (1997). Distributional maps for North American species of tiger beetles (Coleoptera: Cicindelidae). Cicindela.

[ref-45] Pearson DL, Cassola F (1992). World-wide species richness patterns of tiger beetles (Coleoptera: Cicindelidae): indicator taxon for biodiversity and conservation studies. Conservation Biology.

[ref-46] Pearson DL, Cassola F (2005). A quantative analysis of species descriptions of tiger beetles (Coleoptera: Cicindelidae), from 1978 to 2004, and notes about related developments in biodiversity studies. Coleopterologists Bulletin.

[ref-47] Pearson DL, Vogler AP (2001). Tiger beetles: the evolution, ecology and diversity of the cicindelids.

[ref-48] Pimm SL, Brown JH (2004). ECOLOGY: domains of diversity. Science.

[ref-49] Putchkov AV, Matalin AV, Löbl L, Smetana A (2003). Subfamily Cicindelinae Latreille, 1802. Catalogue of Palaearctic Coleoptera V.1. Archeostemata-Myxophaga–Adephaga.

[ref-50] Rewicz T, Jaskuła R (2018). Catch fast and kill quickly: do tiger beetles use the same strategies when hunting different types of prey?. PeerJ.

[ref-51] Rodríguez JP, Pearson DL, Barrera RR (1998). A test for the adequacy of bioindicator taxa: are tiger beetles (Coleoptera: Cicindelidae) appropriate indicators for monitoring the degradation of tropical forests in Venezuela?. Biological Conservation.

[ref-52] Rodríguez-Flores PC, Gutiérrez-Rodríguez J, Aguirre-Ruiz EF, García-París M (2016). Salt lakes of La Mancha (Central Spain): a hot spot for tiger beetle (Carabidae, Cicindelinae) species diversity. ZooKeys.

[ref-53] Satoh A, Hori M (2005). Microhabitat segregation in larvae of six species of coastal tiger beetles in Japan. Ecological Research.

[ref-54] Satoh A, Uéda T, Ichion E, Hori M (2006). Distribution and habitat of three species of riparian tiger beetle in the Tedori River System in Japan. Environmental Entomology.

[ref-55] Schultz TD (1989). Habitat preferences and seasonal abundances of eight sympatric species of tiger beetle, genus *Cicindela* (Coleoptera: Cicindelidae), in Bastrop State Park, Texas. Southwestern Naturalist.

[ref-56] Schultz TD, Hadley NF (1987). Microhabitat segregation and physiological differences in co-occurring tiger beetle species, *Cicindela oregona* and *Cicindela tranquebarica*. Oecologia.

[ref-66] Šekeroğlu E, Aydõn G (2002). Distribution and habitats of the tiger beetle *Megacephala euphratica* in the Çukurova Delta, southern Turkey (Coleoptera: Cicindelidae). Zoology in the Middle East.

[ref-57] Ter Braak CJF, Šmilauer P (2002). CANOCO reference manual and canodraw for windows user’s guide: software for canonical community ordination (Version-4.5).

[ref-58] Thompson JD (2005). Plant evolution in the Mediterranean.

[ref-59] Willig MR, Kaufmann DM, Stevens RD (2003). Latitudinal gradients of biodiversity: pattern, process, scale and synthesis. Annual Review of Ecology and Systematics.

[ref-60] Willis HL (1967). Bionomics and zoogeography of tiger beetles of saline habitats in the central United States (Coleoptera: Cicindelidae). University of Kansas Science Bulletin.

[ref-61] Yanko-Hombach V, Gilbert AS, Panin N, Dolukhanov PM (2006). The Black Sea flood question: changes in coastline, climate and human settlement.

[ref-62] Zerm M, Adis J (2001). Spatio-temporal distribution of larval and adult tiger beetles (Coleoptera: Cicindelidae) from open areas in Central Amazonian floodplains (Brazil). Studies on Neotropical Fauna and Environment.

[ref-63] Zettel H, Wiesner J (2018). *Cylindera* (*Conidera*) *mindoroana* sp. n. a new tiger beetle species from the Philippines. Insecta Mundi.

